# Timing of Treatment with the Flavonoid 7,8-DHF Critically Impacts on Its Effects on Learning and Memory in the Ts65Dn Mouse

**DOI:** 10.3390/antiox8060163

**Published:** 2019-06-06

**Authors:** Andrea Giacomini, Fiorenza Stagni, Marco Emili, Beatrice Uguagliati, Roberto Rimondini, Renata Bartesaghi, Sandra Guidi

**Affiliations:** 1 Department of Biomedical and Neuromotor Sciences, University of Bologna, 40126 Bologna, Italy; andrea.giacomini7@unibo.it (A.G.); fiorenza.stagni2@unibo.it (F.S.); marco.emili2@unibo.it (M.E.); beatrice.uguagliati@unibo.it (B.U.); 2 Department of Medical and Surgical Sciences, University of Bologna, 40126 Bologna, Italy; roberto.rimondini@unibo.it

**Keywords:** Down syndrome, Ts65Dn mouse, Cognitive impairment, Therapy, Flavonoids, 7,8-dihydroxyflavone, Learning and memory

## Abstract

No therapies currently exist for intellectual disability in Down syndrome (DS). In view of its similarities with DS, including learning and memory (L&M) defects, the Ts65Dn mouse model of DS is widely used for the design of therapy. 7,8-dihydroxyflavone (7,8-DHF), a flavonoid that targets the tropomyosin-related kinase B (TrkB) receptor of brain-derived neurotrophic factor (BDNF), exerts positive effects in various brain disease models. Based on previous demonstration that administration of 7,8-DHF in the postnatal period P3-P15 restores hippocampal neurogenesis and spinogenesis, we sought to establish whether these effects translate into behavioral benefits after treatment cessation. We found that Ts65Dn mice treated with 7,8-DHF (5.0 mg/kg/day) during postnatal days P3-P15 did not show any L&M improvement at one month after treatment cessation, indicating that the effects of 7,8-DHF on the brain are ephemeral. Based on evidence that chronic treatment with 7,8-DHF in juvenile Ts65Dn mice restores L&M, we sought to establish whether a similar effect is elicited in adulthood. We found that Ts65Dn mice treated with 7,8-DHF (5.0 mg/kg/day) for about 40 days starting from 4 months of age did not show any improvement in L&M. The results suggest that timing of therapy with 7,8-DHF is a critical issue for attainment of positive effects on the brain.

## 1. Introduction

Down syndrome (DS), a relatively high-incidence (1:750/1000) genetic disorder due to triplication of chromosome 21 (Chr21), is characterized by various medical problems and intellectual disability (ID). The gene burden, due to triplication of Chr21, disrupts a number of developmental processes, including brain development. Although the severity of ID may be variable, in most cases, individuals with DS cannot lead an autonomous life. Thus, ID represents a concern for families and society. Regarding the causes of ID, neurogenesis reduction during the earliest life stages and impaired dendritic development are the factors that most likely play a prominent role (see [1,2]). Unfortunately, no therapies are currently available for the amelioration of ID in individuals with DS. 

A recent study has shown that it is possible to inactivate one of the three Chr21 in vitro and that deficits in proliferation can thus be reversed [3]. Although this opens new avenues, the possibility of a “chromosome therapy” in vivo is, at the moment, very remote. It ensues that attempts to correct the developmental defects due to trisomy must necessarily act downstream of genes and target molecular pathways that are involved in the developmental alterations of DS. Since the processes of neurogenesis and dendritogenesis are under the regulation of various signaling pathways, it may be possible to take advantage of this manifold control and attempt to correct neurogenesis and dendritogenesis defects in DS with a variety of agents. By exploiting the Ts65Dn mouse, one of the most popular models of DS [4], we and others found that different pharmacological interventions in the early neonatal period or in the embryonic period can rescue neurogenesis, dendritic development, and behavior (see [5]). It must be noted that some of these studies remain at the level of “proof of principle” because the drugs used, in view of their chemical nature, may have a limited translational impact. 

Flavonoids are natural substances belonging to the large class of polyphenols that are present in fruits and vegetables. Certain polyphenols may have considerable neuroprotective effects in different brain pathologies, including neurodegenerative diseases [6]. Among polyphenols, flavonoids have been shown to exert beneficial effects in a variety of functions and organs, including the brain, in health, and in disease [7,8]. The mechanisms whereby flavonoids exert their effects are thought to depend largely on their antioxidant properties. Flavonoids (and other polyphenols), however, may also interact with neuronal receptors and kinase signaling pathways, thereby modulating certain cellular processes [6,9]. This makes the use of flavonoids as therapeutic agents particularly attractive because they may exert beneficial effects on the brain through their antioxidant actions as well as by acting through specific signaling cascades. The flavonoid 7,8-dihydroxyflavone (7,8-DHF) specifically binds to the tropomyosin-related kinase receptor B (TrkB) and mimics the effects of brain-derived neurotrophic factor (BDNF), its natural ligand. BDNF is a neurotrophin that plays a key role in brain development and is under-expressed in the fetal DS brain and in the brain of the Ts65Dn mouse [10,11,12,13,14,15]. Therapy with BDNF is unfeasible due to its poor blood-brain-barrier (BBB) penetration. The flavonoid 7,8-DHF, however, can cross the BBB and, by binding to the TrkB receptor, can mimic the action of BDNF [16]. In view of the safe profile of flavonoids, and thus of their good translational impact, in a recent study we examined the effects of a therapy with 7,8-DHF on neurogenesis and dendritic development in the Ts65Dn mouse. Results showed that a short treatment from postnatal day 3 (P3) to P15 was sufficient to restore neurogenesis and dendritic spine development in the hippocampal dentate gyrus, a region that produces most of its neurons in the first postnatal period, and that treatment from P3 to P45 restored hippocampus-dependent learning and memory (L&M) [17].

A key question in the field of pharmacotherapies for DS regards the duration of treatment. An ideal treatment would be one that corrects the brain defects of DS and whose effects are retained after treatment cessation. While in animal models this issue may be of limited relevance, in the case of individuals with DS, and above all children, the necessity of chronic therapy may be a matter of concern, even in the case of drugs with a safe profile, because the necessity of continuous treatment may pose practical problems. On the basis of these evidences, the first aim of our study was to ascertain whether neonatal treatment with 7,8-DHF is sufficient to induce long-lasting effects on hippocampus-dependent L&M. While it is true that the neonatal period is an ideal time window for the pharmacological rescue of hippocampal development, therapies at later life stages may also exert some benefits because the hippocampus exhibits a good degree of plasticity in adulthood. The second goal of this study was to establish whether treatment with 7,8-DHF at adult life stages improves hippocampus-dependent L&M, similarly to treatment at juvenile life stages.

## 2. Methods

### 2.1. Colony

Ts65Dn mice were obtained by mating B6EiC3Sn a/A-Ts(17^16)65Dn females with C57BL/6JEiJ x C3H/HeSnJ (B6EiC3Sn) F1 hybrid males. Jackson Laboratories (Bar Harbor, ME, USA) provided this parental generation. We used here mice of the first generation of breeding in order to maintain the original genetic background. Genotyping was carried out as previously described [18]. Since C3H/HeSnJ mice have a recessive mutation that causes retinal degeneration, we carried out screening of this gene by standard polymerase chain reaction. Mice that carried a recessive mutation that leads to retinal degeneration were excluded from the study. We considered the day of birth as P0. The veterinary service controlled the animals’ health and well-being. The animals lived in a room with a 12:12 h light/dark cycle and had access to water and food *ad libitum*. This study was carried out in compliance with the European Communities Council Directive of 24 November 1986 (86/609/EEC) for the use of experimental animals and were approved by Italian Ministry of Public Health (813/2016-PR). All efforts were made to reduce animal suffering and to minimize the number of animals.

### 2.2. Experimental Protocol

#### 2.2.1. Experiment 1

**Figure 1:**
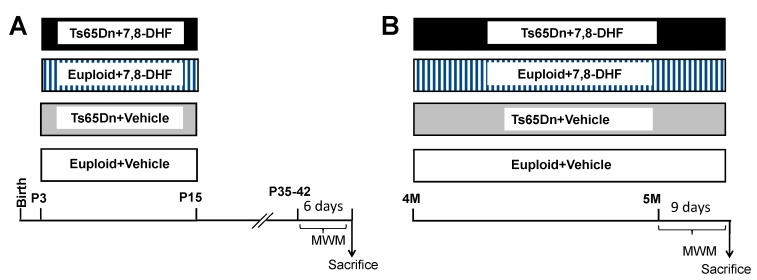
Experimental protocol. (**A**): Euploid and Ts65Dn mice were daily injected with either vehicle (Eu + Vehicle; Ts65Dn + Vehicle) or 5.0 mg/kg of 7,8-DHF (Eu + 7,8-DHF; Ts65Dn + 7,8-DHF) from P3 to P15. Starting from the age of 35-42 days, mice were behaviorally tested with the MWM for 6 days. (**B**): Euploid and Ts65Dn mice were daily injected with either vehicle (Eu + Vehicle; Ts65Dn + Vehicle) or 5.0 mg/kg of 7,8-DHF (Eu + 7,8-DHF; Ts65Dn + 7,8-DHF) starting from 4 months of age. At the age of 5 months mice were behaviorally tested with the MWM for 9 days. Treatment was continued during behavioral testing. Abbreviations: 7,8-DHF, 7,8-dihydroxyflavone; M, months; MWM; Morris Water Maze; P, postnatal.

Euploid and Ts65Dn mice were daily subcutaneously injected (at 9–10am) with 7,8-DHF (5.0 mg/kg in vehicle) or vehicle (PBS with 1% DMSO) from P3 to P15. This timing was selected based on a protocol used in a previous study by our group to examine the long-term outcome of other treatments in Ts65Dn pups [12,19,20]. Mice injected with 7,8-DHF will be called “treated mice” (*n* = 11 treated euploid mice: seven males and four females; *n* = 9 treated Ts65Dn mice: six males and three females). Mice injected with the vehicle will be called “untreated mice” (*n* = 11 untreated euploid mice: seven males and four females; *n* = 10 untreated Ts65Dn mice: six males and four females). Starting from the age of 35–42 days, the behavior of these mice was tested with the Morris Water Maze test (MWM) (Figure 1A).

#### 2.2.2. Experiment 2

Male mice aged 4 months received a daily intraperitoneal injection of 7,8-DHF (5.0 mg/kg) dissolved in the vehicle (*n* = 7 euploid mice; *n* = 7 Ts65Dn mice) or vehicle (*n* = 13 euploid mice; *n* = 11 Ts65Dn mice) for 39 days. Behavioral testing with the MWM was carried out during the last 9 days of treatment (Figure 1B). These mice will be called hereafter adult mice. At the end of behavioral testing mice were killed, and after brain removal the left hemisphere was fixed by immersion in PFA 4% and frozen and the right hemisphere was kept at −80 °C and used for western blotting.

### 2.3. Behavioral Testing

Morris Water Maze (MWM). Previous protocols were used for MWM task [20,21]. The apparatus consisted of a large circular water tank (1.00 m diameter, 50 cm height) with a transparent round escape platform (10 cm^2^). The pool was virtually divided into four equal quadrants identified as northeast, northwest, southeast, and southwest. The tank was filled with tap water at a temperature of 22 ± 1.0 °C up to 0.5 cm above the top of the platform and the water was made opaque with milk. The platform was placed in the tank in a fixed position (in the middle of the southwest quadrant). The pool was placed in a large room with various intra- and extra-maze visual cues (squares, triangles, circles and stars). Individual mice were tested in one session of four trials on the first day and in two sessions of 4 trials (inter-session interval of 40 min) in the following 4 days, in Experiment 1, and in the following 7 days, in Experiment 2. Recording was carried out through a video camera located above the pool center and connected to a video tracking system (Ethovision 3.1; Noldus Information Technology B.V., Wageningen, Netherlands). Each mouse was released from one of the following points: North, South, East, or West and could search the platform for up to 60 s. If a mouse was unable to find the platform, it was placed on the platform and remained there for 15 s. In the intervals (10 s) between trials mice were put in an empty cage. The latency to find the hidden platform, proximity to the platform, and swimming speed were evaluated for the learning phase. Retention was evaluated with a single trial (probe trial) 24 h after the last acquisition trial (day 6, in Experiment 1 and day 9, in Experiment 2). The same starting point was used for all mice. Mice could search for the platform for up to 60 s. For the probe trial the following parameters were considered: the latency of the first entrance in the former platform zone, the frequency of entrances in the former quadrant, the proximity to the former platform position (Gallagher’s test), and the percentage of time spent at the periphery (thigmotaxis). In addition, swimming speed was evaluated. All experimental sessions were carried out between 9.00am and 5.00pm. 

### 2.4. DCX Immunohistochemistry

The frozen hemispheres of mice subjected to adult treatment were processed for Doublecortin (DCX) immunohistochemistry (untreated euploid mice: *n* = 6; untreated Ts65Dn mice: *n* = 6; treated euploid mice: *n* = 4; treated Ts65Dn mice: *n* = 4). The brains were cut with a freezing microtome into 30-μm-thick coronal sections that were serially collected in anti-freezing solution (30% glycerol; 30% ethylen-glycol; 10% PBS10X; 0.02% sodium azide; MilliQ to volume). One out of six free-floating sections from the hippocampal formation (*n* = 10 sections) were processed for DCX immunohistochemistry, as previously described [22]. Evaluation of DCX-positive cells in the granule cell layer of the dentate gyrus was carried out in every 6th section using a fluorescence microscope (Nikon Eclipse TE 2000-S inverted microscope; Nikon Corp., Kawasaki, Japan; objective: x 20, 0.50 NA; final magnification: x 200) that was equipped with a Nikon digital camera DS 2MBWc. DCX-positive cells were counted over the entire length of the granule cell layer and their number was expressed as number of cells for 100 µm of linear length.

### 2.5. Western Blotting

In homogenates of the hippocampal formation of adult mice, total proteins were obtained as previously described [23]. We evaluated the levels of (i) TrkB full length (TrkB-FL), using as primary antibody rabbit monoclonal 1:1000 (Cell Signaling Technology, Danvers, MA, USA; 80E3) and as secondary antibody HRP-conjugated anti-rabbit 1:10000 (Jackson Immunoresearch, West Grove, PA, USA; 111-035-003); (ii) phosphorylated TrkB (p-TrkB^Tyr816^), using as primary antibody rabbit polyclonal 1:1000 (Millipore, Burlington, MA, USA; ABN1381) and a secondary antibody HRP-conjugated anti-rabbit 1:10000 (Jackson Immunoresearch, West Grove, PA, USA; 111-035-003); (iii) glyceraldheyde 3-phosphate dehydrogenase (GAPDH), using as primary antibody rabbit polyclonal 1:5000 (Sigma-Aldrich, Saint Louis, MO, USA; G9545) and as secondary antibody HRP-conjugated anti-rabbit 1:10000 (Jackson Immunoresearch, West Grove, PA, USA; 111-035-003). Densitometric analysis of digitized images with ChemiDoc XRS+ was carried out with Image Lab software (Bio-Rad Laboratories, Hercules, CA, USA) and the intensity of each band was normalized to the intensity of the corresponding GAPDH band. 

### 2.6. Statistical Analysis

The reported results are mean ± standard error of the mean (SE). The IBM SPSS 22.0 (International Business Machines Corporation, Armonk, NY, USA) software was used for data analysis. For each variable, data distribution and homogeneity of variances were checked using the Shapiro-Wilk test and Levene’s test respectively, before running statistical analyses. Statistical analysis of the learning phase of the MWM test was carried out using a three-way mixed ANOVA, with genotype (euploid, Ts65Dn) and treatment (vehicle, 7,8-DHF) as grouping factors and days as a repeated measure, followed by the *post hoc* Fisher’s Least Significant Difference (LSD) test. Statistical analysis of the probe test of the MWM, the number of DCX-positive cells, and the levels of the TrkB receptor was carried out with a two-way ANOVA with genotype and treatment as factors, followed by the *post hoc* Fisher LSD test. Table 1 and Table 2 report the p values of the *post hoc* Fisher LSD test for each analysis of the learning phase. A probability level of *p* ≤ 0.05 was considered to be statistically significant. 

## 3. Results

### 3.1. Long-Term Effects of Neonatal Treatment with 7,8-DHF on Hippocampus-Dependent Learning and Memory

In a previous study we found that neonatal administration of 7,8-DHF restores neurogenesis and dendritic spine development in the hippocampus of Ts65Dn pups [17]. In order to establish whether these effects translate into a long-term improvement of hippocampus-dependent L&M, euploid and Ts65Dn mice were treated from P3 to P15 and their behavior was examined at one month after treatment cessation. We used the MWM test because it is a well-established test to assess the effects of genotype and/or treatment on L&M in trisomic mice. After a learning phase of 5 days, on day 6 mice were subjected to the probe test. For the learning phase, we evaluated escape latency and proximity to the platform zone. We additionally evaluated swimming speed, in order to establish whether possible speed differences may affect the outcome of the MWM test. All variables were analyzed with a three-way mixed ANOVA followed by *post hoc* Fisher LSD test. Results of ANOVA are reported in the text and the *post hoc* test results are summarized in Table 1.

**Figure 2:**
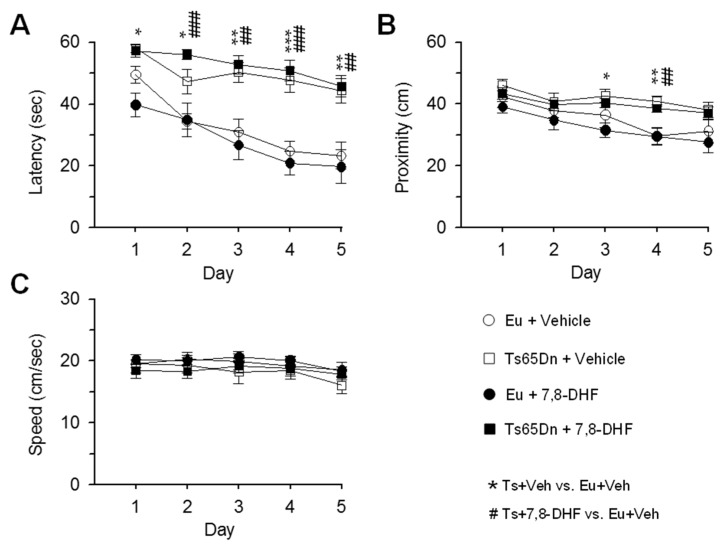
Effect of neonatal treatment with 7,8-DHF on spatial learning in Ts65Dn and euploid mice. Mice were injected with either vehicle or 7,8-DHF in the period P3–P15 and their behavior was tested with the MWM beginning from the age of 35–42 days (untreated euploid mice: *n* = 11; untreated Ts65Dn mice: *n* = 10; treated euploid mice: *n* = 11; treated Ts65Dn mice: *n* = 9). The data in (**A**–**C**) refer to euploid mice that received either vehicle (empty circle) or 7,8-DHF (filled circle) and Ts65Dn mice that received either vehicle (empty square) or 7,8-DHF (filled square). A,B: In the learning phase of the MWM, the latency to reach the platform (**A**), proximity to the platform zone (**B**), and swimming speed (**C**) were evaluated. Values are mean ± standard error (SE). The symbol * indicates a difference between untreated euploid and untreated Ts65Dn mice and the symbol # indicated a difference between untreated euploid mice and treated Ts65Dn. One symbol: *p* ≤ 0.05; two symbols: *p* ≤ 0.01; three symbols: *p* ≤ 0.001 (Fisher LSD test after ANOVA). Abbreviations: 7,8-DHF, 7,8-dihydroxyflavone; cm, centimeters; Eu, euploid; sec, seconds; Ts, Ts65Dn; Veh, vehicle.

A three-way mixed ANOVA on escape latency, with genotype and treatment as grouping factors and day as a repeated measure revealed no effect of genotype x treatment x day. We found no genotype x day interaction, no treatment x day interaction, no genotype x treatment interaction, a main effect of genotype [F(1,37) = 64.79, *p* < 0.001], no main effect of treatment, and a main effect of day [F(4,148) = 16.65, *p* < 0.001]. Unlike untreated euploid mice that exhibited a rapid learning improvement with time, untreated Ts65Dn mice showed a very modest learning enhancement (Figure 2A, Table 1). Treated Ts65Dn mice showed no learning amelioration and their performance remained similar to that of untreated Ts65Dn mice (Figure 2A, Table 1). In treated euploid mice the latency did not undergo changes in comparison with their untreated counterparts (Figure 2A; Table 1). 

A three-way mixed ANOVA on proximity to the former platform position, with genotype and treatment as grouping factors and day as a repeated measure revealed no genotype x treatment x day interaction, no genotype x day interaction, no treatment x day interaction, no genotype x treatment interaction, a main effect of genotype [F(1,30) = 19.81, *p* < 0.001], no main effect of treatment, and a main effect of day [F(4,148) = 15.23, *p* < 0.001]. Unlike untreated euploid mice that exhibited a reduction in the distance from the platform position from day 1 to day 5, untreated Ts65Dn mice underwent no improvement (Figure 2B). Treated Ts65Dn swam at a distance from the platform position similar to that of their untreated counterparts (Figure 2B, Table 1). In treated euploid mice the proximity did not undergo changes in comparison with their untreated counterparts (Figure 2B; Table 1).

A three-way mixed ANOVA on the swimming speed, with genotype and treatment as grouping factors and day as a repeated measure revealed no genotype x treatment x day interaction. We found no genotype x day interaction, no treatment x day interaction, no genotype x treatment interaction, no main effect of genotype, no main effect of treatment, but a main effect of day was detected [F(4,148) = 5.45, *p* < 0.001]. A *post hoc* Fisher LSD test showed no differences in swimming speed between groups, even if on day 5 untreated Ts65Dn mice exhibited a slightly lower speed in comparison with the other groups (Figure 2C, Table 1).

As an index of spatial memory in the probe test we evaluated the following variables: (i) latency to enter the former platform zone (latency); (ii) frequency of entrances into the former quadrant (frequency); (iii) proximity to the former platform position (Gallagher’s test; proximity); (iv) percentage of time spent at the periphery (thigmotaxis). In addition, we evaluated swimming speed.

**Figure 3:**
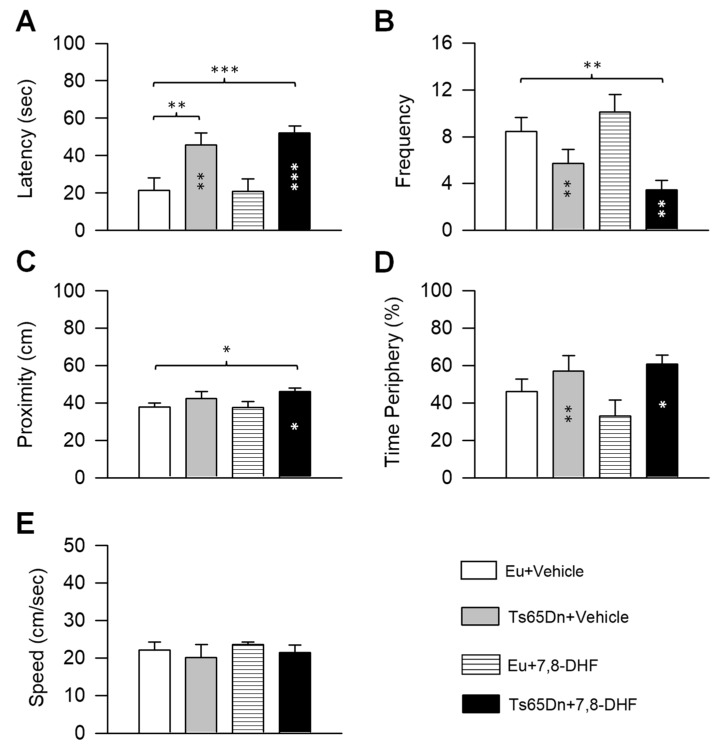
Effect of neonatal treatment with 7,8-DHF on spatial memory in Ts65Dn and euploid mice. Memory was assessed in the probe test as latency to reach the former platform zone (**A**), frequency of crossings over the former platform quadrant (**B**), proximity to the former platform zone (**C**), percentage of time spent at the periphery (**D**) (same mice as in Figure 2). (**E**): Swimming speed during the probe test. Values are mean ± SE. * *p* ≤ 0.05; ** *p* ≤ 0.01; *** *p* ≤ 0.001 (Fisher LSD test after ANOVA). Black asterisks in the gray bar indicate a difference between untreated Ts65Dn mice and treated euploid mice; white asterisks in the black bar indicate a difference between treated Ts65Dn mice and treated euploid mice. Abbreviations: 7,8-DHF, 7,8-dihydroxyflavone; cm, centimeters; Eu, euploid; sec, seconds.

A two-way ANOVA on the latency showed no genotype x treatment interaction, a main effect of genotype [F(1,37) = 25.90, *p* < 0.001], and no effect of treatment. *Post hoc* Fisher LSD test revealed that untreated Ts65Dn mice had a longer latency than untreated euploid mice and that in treated Ts65Dn mice the latency did not undergo any reduction (Figure 3A). In treated euploid mice, the latency did not undergo changes in comparison with their untreated counterparts (Figure 3A).

A two-way ANOVA on the frequency showed no genotype x treatment interaction, a main effect of genotype [F(1,37) = 16.85, *p* < 0.001], and no effect of treatment. *Post hoc* Fisher LSD test showed no difference between untreated euploid and Ts65Dn mice, although Ts65Dn mice exhibited a reduced frequency of entrances in comparison with euploid mice. Treated Ts65Dn mice exhibited no increase in the frequency of entrances that remained lower than that of untreated euploid mice (Figure 3B). In treated euploid mice the frequency did not change and remained similar to that of their untreated counterparts (Figure 3B). 

A two-way ANOVA on the proximity showed no genotype x treatment interaction, a main effect of genotype [F(1,37) = 5.76, *p* = 0.022], and no effect of treatment. In untreated and treated Ts65Dn mice the distance from the platform zone was larger in comparison with untreated euploid mice, although the difference was statistically significant for treated Ts65Dn mice only (Figure 3C). In treated euploid mice, the proximity did not change in comparison with their untreated counterparts (Figure 3C).

A two-way ANOVA on the percentage of time spent at the periphery showed no genotype x treatment interaction, a main effect of genotype [F(1,37) = 7.66, *p* = 0.009], and no main effect of treatment. A *post hoc* Fisher LSD test demonstrated that in untreated Ts65Dn mice the percentage of time spent at the periphery was similar to that of untreated euploid mice. Treated Ts65Dn mice showed no difference in the time spent at the periphery in comparison with their untreated counterparts. Both treated and untreated Ts65Dn mice spent more time at the periphery in comparison with treated euploid mice (Figure 3D). The latter finding can be accounted for by the fact that in treated euploid mice the time at the periphery was lower than that of their untreated counterparts, although this difference was not statistically significant (Figure 3D).

A two-way ANOVA on the swimming speed showed no genotype x treatment interaction, no main effect of genotype, and no main effect of treatment. A *post hoc* Fisher LSD test showed no difference between groups (Figure 3E).

These results confirm impairment of L&M in untreated Ts65Dn mice. In treated Ts65Dn mice, the variables analyzed for the learning phase did not show any amelioration day by day. Similarly, in the probe test treated Ts65Dn mice showed a behavior that was similar to that of their untreated counterparts. Taken together, these results show a lack of long-term effect of neonatal treatment with 7,8-DHF on L&M.

### 3.2. Effects of Adult Treatment with 7,8-DHF on Hippocampus-Dependent Learning and Memory

In a previous study, we found that early treatment with 7,8-DHF for approximately 40 days (from P3 to P45) restored L&M in Ts65Dn mice [17]. In the current study we were interested in establishing whether treatment with 7,8-DHF has a similar beneficial effect in adulthood. To this purpose, we treated mice with 7,8-DHF or vehicle beginning from 4 months of age. Behavioral testing with the MWM test started when mice had 5 months and lasted 9 days during which treatment was not interrupted. Since brain plasticity decreases with age and learning may require more time in adulthood than in juvenile life stages, in adult mice we prolonged the learning phase to 8 days in order to give mice more time to learn. On day 9, mice were subjected to the probe test in order to evaluate spatial memory. 

**Figure 4:**
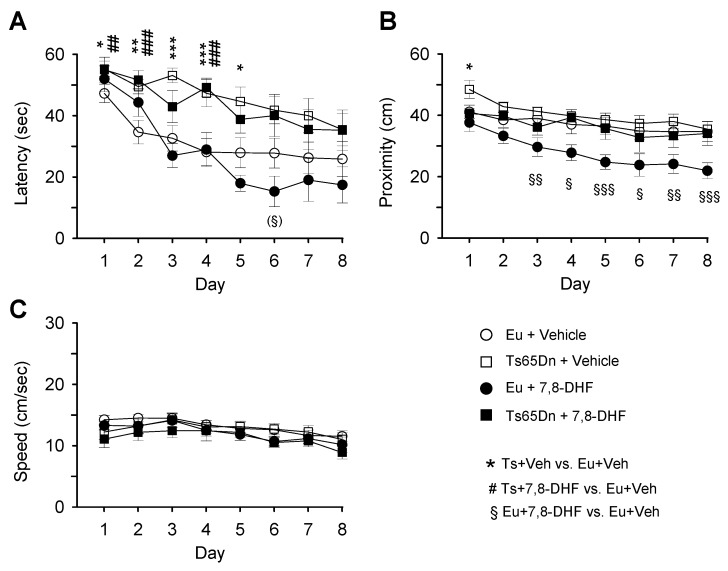
Effect of treatment with 7,8-DHF on spatial learning in adult mice. Euploid and Ts65Dn mice aged 4 months received one injection of vehicle or 7,8-DHF for 39 days and were subjected to the MWM starting from the 30th day of treatment. Spatial learning was assessed with MWM in untreated euploid mice (*n* = 13), untreated Ts65Dn mice (*n* = 11), treated euploid mice (*n* = 7), and treated Ts65Dn mice (*n* = 7). The curves in A-C report data of euploid mice that received either vehicle (empty circle) or 7,8-DHF (filled circle) and Ts65Dn mice that received either vehicle (empty square) or 7,8-DHF (filled square). A,B: In the learning phase of the MWM, the latency to reach the platform (**A**), proximity to the platform zone (**B**), and swimming speed (**C**) were evaluated. Values are mean ± SE. The symbol * indicates a difference between untreated euploid and untreated Ts65Dn mice, the symbol # indicated a difference between untreated euploid mice and treated Ts65Dn mice, and the symbol § indicates a difference between treated and untreated euploid mice. One symbol in brackets: p < 0.06; one symbol: *p* ≤ 0.05; two symbols: *p* ≤ 0.01; three symbols: *p* ≤ 0.001 (Fisher LSD test after ANOVA). Abbreviations: 7,8-DHF, 7,8-dihydroxyflavone; cm, centimeters; Eu, euploid; sec, seconds; Ts, Ts65Dn; Veh, vehicle.

A three-way mixed ANOVA on escape latency, with genotype and treatment as grouping factors and day as a repeated measure revealed no genotype x treatment x day interaction. We found a marginally significant genotype x day interaction [F(7,238) = 2.00, *p* = 0.056], a treatment x day interaction [F(7,238) = 2.23, *p* = 0.032], no genotype x treatment interaction, a main effect of genotype [F(1,34) = 20.63, *p* < 0.001], no main effect of treatment, and a main effect of day [F(7,238) = 24.66, *p* < 0.001]. Unlike untreated euploid mice that underwent a rapid learning amelioration with time, untreated Ts65Dn mice showed a modest learning amelioration (Figure 4A, Table 2). Similarly, treated Ts65Dn mice showed no learning improvement and their performance remained similar to that of untreated Ts65Dn mice (Figure 4A, Table 2). Starting from day 5, treated euploid mice exhibited a latency reduction in comparison with their untreated counterparts (Figure 4A, Table 2), although this difference was not statistically significant. 

A three-way mixed ANOVA on proximity to the former platform position (proximity), with genotype and treatment as grouping factors and day as a repeated measure revealed no genotype x treatment x day interaction, no genotype x day interaction, no treatment x day interaction, no genotype x treatment interaction, a main effect of genotype [F(1,34) = 13.51, *p* = 0.001], no effect of treatment, and a main effect of day [F(4,120) = 14.82, *p* < 0.001]. Figure 4B shows that in untreated euploid mice and treated and untreated Ts65Dn mice the distance from the platform position decreased moderately from day 1 to day 8. A comparison of treated and untreated Ts65Dn mice revealed no effect of treatment. In contrast, treated euploid mice exhibited an increase in proximity starting from day 3 and for all remaining days in comparison with their untreated counterparts (Figure 4B; Table 2).

A three-way mixed ANOVA on the swimming speed, with genotype and treatment as grouping factors and day as a repeated measure revealed no genotype x treatment x day interaction. We found no genotype x day interaction, no treatment x day interaction, no genotype x treatment interaction, no main effect of genotype, no main effect of treatment, and a main effect of day [F(7,238) = 8.49, *p* < 0.001]. No differences in the swimming speed between groups were revealed by a *post hoc* Fisher LSD test (Figure 4C, Table 2).

**Figure 5:**
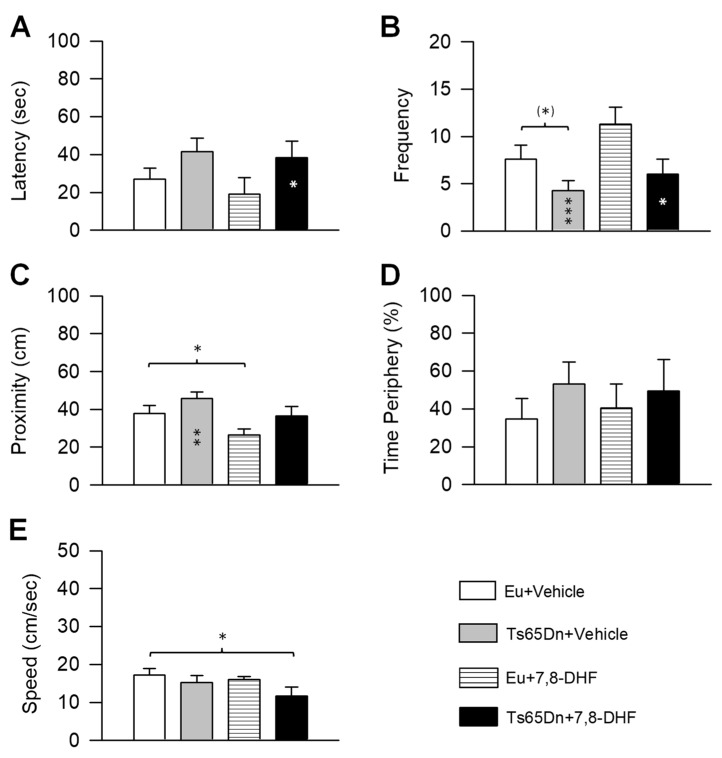
Effect of treatment with 7,8-DHF on spatial memory in adult Ts65Dn and euploid mice. Memory was assessed in the probe test as latency to reach the former platform zone (**A**), frequency of crossings over the former platform quadrant (**B**), proximity to the former platform zone (**C**), percentage of time spent at the periphery (**D**) (same mice as in Figure 4). E: Swimming speed during the probe test. Values are mean ± SE. (*) *p* < 0.06; * *p* ≤ 0.05; ** *p* ≤ 0.01; *** *p* ≤ 0.001; (Fisher LSD test after ANOVA). Black asterisks in the gray bar indicate a difference between untreated Ts65Dn mice and treated euploid mice; white asterisks in the black bar indicate a difference between treated Ts65Dn mice and treated euploid mice. Abbreviations: 7,8-DHF, 7,8-dihydroxyflavone; cm, centimeters; Eu, euploid; sec, seconds.

In the probe test, we analyzed the same variables as in Experiment 1. A two-way ANOVA on the latency showed no genotype x treatment interaction, a main effect of genotype [F(1,32) = 5.59, *p* = 0.024], and no main effect of treatment. *Post hoc* Fisher LSD test showed that untreated Ts65Dn mice had a larger latency in comparison with untreated and treated euploid mice, albeit a statistically significant difference was found in comparison with treated euploid mice only. Treated Ts65Dn mice did not undergo a latency improvement in comparison with their untreated counterparts (Figure 5A). 

A two-way ANOVA on the frequency showed no genotype x treatment interaction, a main effect of genotype [F(1,32) = 6.44, p = 0.016], and no main effect of treatment. *Post hoc* Fisher LSD test revealed a reduced frequency of entrances in untreated Ts65Dn mice in comparison with untreated euploid mice. A frequency improvement was not found in treated Ts65Dn mice in comparison with their untreated counterparts (Figure 5B). 

A two-way ANOVA on the proximity showed no genotype x treatment interaction, a main effect of genotype [F(1,32) = 5.08, *p* = 0.031], and a main effect of treatment [F(1,32) = 6.54, *p* = 0.016]. *Post hoc* Fisher LSD test showed that the distance from the former platform zone was larger in untreated Ts65Dn mice in comparison with untreated and treated euploid mice, albeit a statistically significant difference was found in comparison with treated euploid mice only (Figure 5C). In treated Ts65Dn mice, the distance from the former platform zone remained similar to that of their untreated counterparts (Figure 5C). In contrast, treated euploid mice swam at a closer distance in comparison with untreated euploid mice (Figure 5C). 

A two-way ANOVA on the percentage of time spent at the periphery showed no genotype x treatment interaction, no main effect of genotype, and no main effect of treatment. *Post hoc* Fisher LSD test showed that the percentage of time spent at the periphery was similar in all the experimental groups (Figure 5D). 

A two-way ANOVA on the swimming speed showed no genotype x treatment interaction, no main effect of genotype, and no main effect of treatment. *Post hoc* Fisher LSD test showed a similar swimming speed across groups, save for treated Ts65Dn mice that exhibited a reduced speed in comparison with untreated euploid mice (Figure 5E).

### 3.3. Effects of Adult Treatment with 7,8-DHF on Neurogenesis

**Figure 6:**
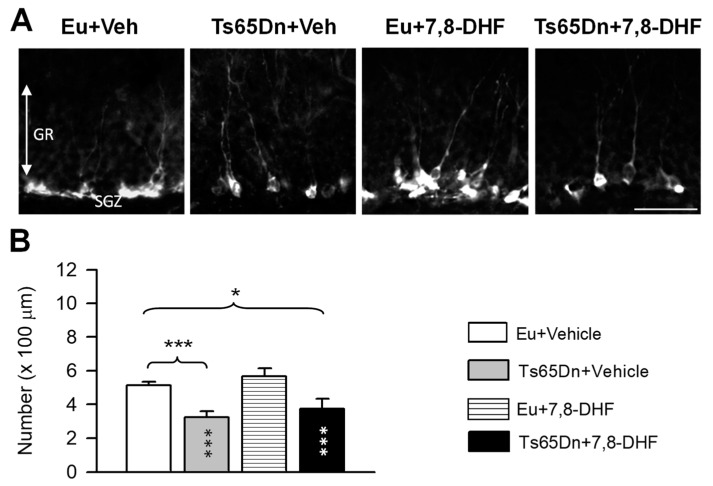
Effect of treatment with 7,8-DHF on the number of new granule neurons in the dentate gyrus. (**A**): Sections processed for fluorescent immunostaining for DCX from the dentate gyrus of animals of each experimental group. Calibration bar: 50 µm. (**B**): Number of DCX-positive cells per unit length of the granule cell layer in untreated euploid (*n* = 6) and Ts65Dn mice (*n* = 6) and euploid (*n* = 4) and Ts65Dn (*n* = 4) mice that received 7,8-DHF. Values are mean ± SE. * *p* < 0.05; *** *p* < 0.001 (Fisher LSD test after two-way ANOVA). Asterisks in the gray bar indicate a difference between untreated Ts65Dn mice and treated euploid mice; white asterisks in the black bar indicate a difference between treated euploid and treated Ts65Dn mice. Abbreviations: 7,8-DHF, 7,8-dihydroxyflavone; Eu, euploid; Gr, granule cell layer; SGZ, subgranular zone; Veh, vehicle.

Doublecortin (DCX) is a microtubule-associated phosphoprotein located in the periphery of the soma with a pattern corresponding to microtubule distribution [24]. In the period of elongation of neuritic processes (1–4 weeks after neuron birth), immature granule cells express DCX which allows estimation of the number of new granule neurons. Hippocampal sections of mice treated for one month with 7,8-DHF or vehicle were processed for immunohistochemistry for DCX in order to clarify possible effects of treatment with 7,8-DHF on hippocampal neurogenesis. A two-way ANOVA on the number of DCX-positive cells showed no genotype x treatment interaction, a significant effect of genotype [F(1,14) = 24.83, *p* < 0.001] but no effect of treatment. Consistently with previous studies, untreated Ts65Dn mice had fewer new granule cells in comparison with untreated euploid mice (Figure 6A,B). In treated Ts65Dn mice there was no increase in the number of new granule neurons in comparison with their untreated counterparts (Figure 6A,B). In euploid mice treatment did not affect the number of new granule cells (Figure 6A,B). 

### 3.4. Effects of Adult Treatment with 7,8-DHF on the TrkB Receptor

**Figure 7:**
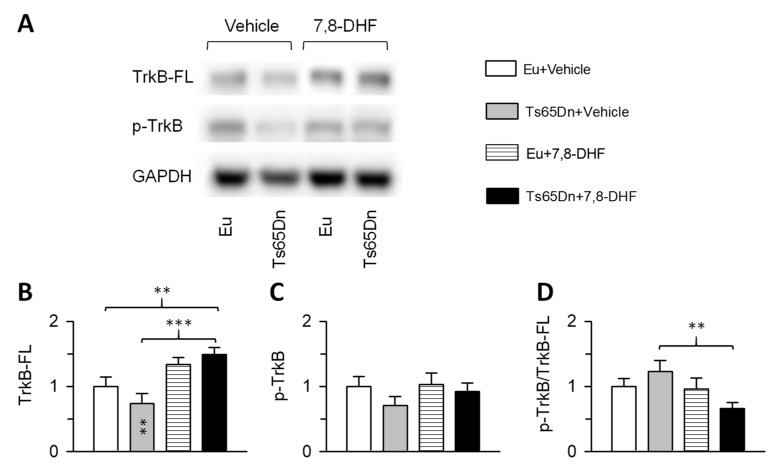
Effects of treatment with 7,8-DHF on the tropomyosin-related kinase receptor B (TrkB) receptor protein levels. (**A**): representative western blots showing immunoreactivity for the phosphorylated TrkB receptor (p-TrkB^Tyr816^), full length TrkB receptor (TrkB-FL), and housekeeping gene GAPDH. B-D: Levels of TrkB-FL (**B**), p-TrkB^Tyr816^ (**C**), and p-TrkB^Tyr816^/TrkB-FL (**D**) of untreated euploid (*n* = 8) and Ts65Dn (*n* = 9) and treated euploid (*n* = 7) and Ts65Dn (*n* = 7) mice. Data are expressed as multiple of untreated euploid mice. Values are mean ± SE. ** *p* < 0.01; *** *p* < 0.001 (Fisher LSD test after two-way ANOVA). Asterisks in the gray bar indicate a difference between untreated Ts65Dn mice and treated euploid mice Abbreviations: 7,8-DHF, 7,8-dihydroxyflavone; Eu, euploid.

Binding of BDNF or its mimetic 7,8-DHF to the TrkB full length receptor (TrkB-FL), causes its autophosphorylation and, hence, activation. Based on previous studies [16,17,25,26,27], we evaluated the activation of the TrkB receptor by examining the phosphorylation levels of Tyr 816. The expression levels and activity of the TrkB receptor were evaluated in hippocampal homogenates of treated and untreated mice through western blot analysis (Figure 7).

A two-way ANOVA on the levels of the TrkB-FL receptor showed no genotype x treatment interaction, no main effect of genotype but a main effect of treatment [F(1,27) = 18.490, *p* < 0.001]. Although untreated Ts65Dn mice had reduced levels of TrkB-FL, *post hoc* Fisher LSD test showed no differences between untreated Ts65Dn and euploid mice (Figure 7B). Treatment with 7,8-DHF caused an increase in the levels of the TrkB-FL receptor in Ts65Dn mice in comparison with their untreated counterparts (Figure 7B). An increase in TrkB-FL receptor levels also took place in treated euploid mice in comparison with their untreated counterparts, although the difference was not statistically significant. A two-way ANOVA on the levels of the phosphorylated form of TrkB receptor (p-TrkB^Tyr816^) showed no genotype x treatment interaction, no main effect of genotype and no main effect of treatment. Although untreated Ts65Dn mice had reduced levels of p-TrkB^Tyr816^, *post hoc* Fisher LSD test showed no differences between untreated Ts65Dn and euploid mice (Figure 7C). Treatment caused no changes in p-TrkB^Tyr816^ levels both in euploid and Ts65Dn mice (Figure 7C). A two-way ANOVA on the ratio between p-TrkB^Tyr816^ and Trkb-FL showed no genotype x treatment interaction, no main effect of genotype but a main effect of treatment [F(1,27) = 5.049, p = 0.033]. *Post hoc* Fisher LSD test showed no differences between groups save for treated Ts65Dn mice that underwent a reduction in comparison with their untreated counterparts (Figure 7D).

## 4. Discussion

### 4.1. Neonatal Treatment with 7,8-DHF Fails to Induce Long-Lasting Effects on Hippocampus-Dependent Learning and Memory in Ts65Dn Mice

Results show that Ts65Dn mice neonatally-treated with 7,8-DHF for 13 days do not exhibit any improvement in their defective L&M performance at approximately one month after treatment cessation. The bulk of the granule neurons forming the dentate gyrus are born within the first two postnatal weeks, a period that also corresponds to prominent dendritic maturation of the granule neurons [28,29,30,31]. We previously found that 13 days of neonatal treatment with 7,8-DHF fully rescues the number of hippocampal granule neurons and granule neuron dendritic spine density [17]. Thus, restoration of the hippocampal development during the most critical period of hippocampal neurogenesis and neuron maturation may be expected to result in long-lasting effects on hippocampal function. Indeed, previous studies showed that neonatal treatment in the period P3–P15 with the antidepressant fluoxetine and the gamma-secretase inhibitor ELND006 generated effects that outlasted treatment cessation and were retained when mice were aged 45 days [12,32]. Similarly, in Ts65Dn mice embryonic treatment with fluoxetine has effects that extend to adult life stages, as does embryonic/early postnatal choline supplementation [33,34,35]. In addition, embryonic treatment with a synthetic inhibitor of Dual specificity Tyrosine(Y) Regulated Kinase 1A (Dyrk1A) prevents postnatal learning defects in the Ts1Cje model of DS [36]. On the other hand, neonatal treatment with the flavonoid epigallocatechin-3-gallate (EGCG) in the period P3–P15 restores hippocampal development but this effect does not result in any improvement in hippocampus-dependent memory at one month after treatment cessation [20]. The comparison of these findings suggests that, unlike other compounds, the flavonoids 7,8-DHF and EGCG (and, possibly, flavonoids in general) are unable to exert long-lasting effects on the brain. 

Taken together the results of the current and previous studies prompt a series of considerations. (i) The flavonoid 7,8-DHF may represent an appropriate drug for DS because continuous treatment from P3 to P45 rescues hippocampus-dependent L&M [17]. (ii) The lack of long-term effects of 7,8-DHF, however, suggests that it is unable to permanently revert the brain alterations that characterize DS. (iii) Since the effects of 7,8-DHF on hippocampal development are ephemeral, continuous treatment appears to be necessary in order to maintain the hippocampus in its restored state. (iv) There are drugs such as fluoxetine and ELND006 that engender effects that are retained after treatment discontinuation. This suggests that it is possible to pharmacologically restore the aberrant cellular processes in DS in a permanent manner. It is worth noting that the effects of neonatal treatment with fluoxetine are retained at about three months after treatment cessation [37]. (v) Following the previous considerations, treatments for DS might be categorized as “symptomatic”, if they exert transitory effects on the trisomic brain and “curative” if they are able to permanently readjust cellular processes that are altered in DS. 

We have used 7,8-DHF in order to improve hippocampal development in Ts65Dn mice because it is a mimetic of BDNF. This neurotrophin is important for brain development and is under-expressed in the trisomic brain. EGCG is a selective inhibitor of DYRK1A, a kinase over-expressed in DS that is an important determinant of neurogenesis impairment in DS. It must be observed that both 7,8-DHF and EGCG, similarly to other flavonoids, exert antioxidant activity [38,39] and that their beneficial effects may also be due to their antioxidant actions. This idea is particularly reasonable in the case of DS because mitochondrial dysfunction, a typical feature of DS, may also be involved in neurogenesis failure and neurodegeneration. Interestingly, the antioxidant action of 7,8-DHF counteracts chemically-induced cytotoxicity by preventing cell death and mitochondrial dysfunction [39]. A recent study [40] showed alteration of mitochondrial bioenergetics during proliferation in cultures of neural progenitor cells from the hippocampus of Ts65Dn mice. Interestingly, exposure to EGCG restored the efficiency of oxidative phosphorylation and mitochondrial biogenesis, and improved proliferation, with a mechanism that was DYRK1A-independent. This evidence suggests the possibility that an antioxidant action may contribute to the beneficial effects exerted by 7,8-DHF and EGCG in the trisomic condition. In addition, it has been shown that 7,8-DHF suppresses GABAergic inhibition [41]. Because GABAergic inhibition is increased in Ts65Dn mice [42], suppression of inhibition by 7,8-DHF (and, possibly, other flavonoids) may contribute to the overall beneficial effects of 7,8-DHF in DS. 

The current study clearly shows that, whatever the mechanism or mechanisms involved in the improvement of hippocampal development, 7,8-DHF is unable to induce permanent effects. However, these apparently negative results must be considered from a broader perspective. Flavonoids are compounds present in fruits and vegetables that have been used by humankind as natural remedies for a variety of diseases. 7,8-DHF, by functioning as an agonist of the TrkB receptor as well as an antioxidant, may exert various benefits on the brain, keeping it healthy and, at adult life stages, preventing neurodegeneration [43]. Thus, it is not unreasonable to hypothesize that continuous or semi-continuous treatment with 7,8-DHF may represent a therapy that is potentially usable in individuals with DS without adverse effects. Further studies are needed aimed at individuating in the Ts65Dn mouse optimum treatment schedules in terms of duration and time interval between repeated treatments.

### 4.2. Adult Therapy with 7,8-DHF Does Not Replicate the Effects of Therapy in Young Ts65Dn Mice

In the current study, we were interested in establishing whether therapy with 7,8-DHF can improve/restore spatial learning and memory, as assessed with the MWM test, in adult Ts65Dn mice. We used a dose of 5.0 mg/kg/day, because this dose is the most effective in Ts65Dn pups, restores L&M in young Ts65Dn mice, and induces positive effects in various disease models [16,25,27,44,45,46,47,48,49]. We found, however, that adult Ts65Dn mice treated for approximately 40 days with 7,8-DHF showed no sign of improvement of spatial L&M. In contrast, euploid mice subjected to the same treatment showed some benefits in L&M, indicating that the regimen used is at least partially effective in wild-type mice. 

Neurogenesis impairment is thought to be a major determinant of L&M deficits in Ts65Dn mice. Accordingly, it has been shown that treatments that rescue hippocampal neurogenesis in Ts65Dn mice are associated with a behavioral improvement [5]. We found here that treatment with 7,8-DHF did not enhance hippocampal neurogenesis in Ts65Dn mice. The lack of a pro-neurogenic effect of treatment may explain the lack of a behavioral improvement in treated Ts65Dn mice. An analysis of the TrkB-FL receptor showed that in treated Ts65Dn mice there was an increase in its levels in comparison with their untreated counterparts. While this result is consistent with previous evidence in an Alzheimer disease mouse model [44], it is at variance with other studies that failed to detect an increase in the levels of TrkB-FL [26,27,50]. Our previous study in Ts65Dn mice [17] and studies in other mouse models [16,25,26,27] have shown that treatment with 5.0 mg/kg/day of 7,8-DHF enhances the phosphorylation (i.e., activation) of the TrkB receptor at Tyr 816 and induces behavioral benefits. Unlike in our previous study, however, we found here that treatment with 5.0 mg/kg/day of 7,8-DHF did not increase the levels of p-TrkB^Tyr816^. It must be noted that in our previous study, Ts65Dn mice were treated in the neonatal period [17], while in the current study mice were treated in adulthood. A recent study shows that adult Ts65Dn mice treated with 20 mg/kg/day of 7,8-DHF for 30 days exhibit an increase in the levels of p-TrkB^Tyr817^ (which is equivalent to mouse/rat Tyr-816) [51] in the hippocampus (but not in the cortex) [50]. The discrepancy in the effect of treatment on the phosphorylation levels of the TrkB receptor suggests that, unlike in young Ts65Dn mice, a relatively high dose of 7,8-DHF is needed in order to activate the TrkB receptor in adulthood.

The lack of a phosphorylation increase of the TrkB receptor observed here might explain the absence of an improvement in neurogenesis and L&M, as assessed with the MWM. In the study by Parrini et al. [50] Ts65Dn mice underwent restoration of contextual memory (assessed with the contextual fear conditioning test) and novelty discrimination (assessed with the novel object recognition test) [50]. It must be noted that the tests used to evaluate cognitive performance, such as the contextual fear conditioning, novel object recognition, and MWM tests, may exhibit a different sensitivity to treatment. This may contribute to explain the discrepancy between the current results and those obtained in the study by Parrini et al. [50]. Taken together, these findings suggest that in adult Ts65Dn mice, unlike young mice, a relatively high dose of 7,8-DHF is needed in order to obtain a behavioral improvement in spatial L&M. 

## 5. Conclusions

Accumulating evidence points to 7,8-DHF as a promising strategy for different types of brain disorders [43]. Our previous study showed that 7,8-DHF restores L&M in the Ts65Dn model of DS [17]. Based on the observation that 7,8-DHF has no toxic effects in wild-type [43] as well as Ts65Dn [17] mice, therapy with 7,8-DHF may represent an alternative strategy to other drugs, with a good translational value for a brain development in DS. However, our current results show that a brief neonatal treatment is insufficient to engender long-lasting effects on behavior and that a dosage that at juvenile life stages is sufficient to restore memory is insufficient in adulthood. We hope that this information may be useful to the scientific community interested in therapy for DS and other brain disorders. We believe that awareness of the critical issues of the timing of treatment and age dependence of the effective dose of 7,8-DHF is fundamental for the design of appropriate treatment schedules. 

## Figures and Tables

**Table 1:** *p* values of the Fisher LSD test for the indicated variables, during the learning phase of the MWM in euploid and Ts65Dn mice treated with vehicle or 7,8-DHF in the period P3–P15.

-		**Latency (s)**				
		**D1**	**D2**	**D3**	**D4**	**D5**
Eu + Veh	Eu + 7,8-DHF	**0.007**	0.789	0.177	0.207	0.236
	Ts65Dn + Veh	**0.035**	**0.044**	**0.007**	**<0.001**	**0.008**
	Ts65Dn + 7,8-DHF	0.066	**0.001**	**0.003**	**<0.001**	**0.006**
Eu + 7,8-DHF	Ts65Dn + Veh	**<0.001**	**0.024**	**<0.001**	**<0.001**	**<0.001**
	Ts65Dn + 7,8-DHF	**<0.001**	**<0.001**	**<0.001**	**<0.001**	**<0.001**
Ts65Dn + Veh	Ts65Dn + 7,8-DHF	0.823	0.129	0.664	0.575	0.829
-		**Proximity (cm)**				
		**D1**	**D2**	**D3**	**D4**	**D5**
Eu + Veh	Eu + 7,8-DHF	0.565	0.647	0.110	0.471	0.209
	Ts65Dn + Veh	0.062	0.204	**0.045**	**0.003**	0.098
	Ts65Dn + 7,8-DHF	0.270	0.169	0.100	**0.006**	0.146
Eu + 7,8-DHF	Ts65Dn + Veh	**0.017**	0.090	**0.001**	**<0.001**	**0.006**
	Ts65Dn + 7,8-DHF	0.103	0.074	**0.003**	**0.001**	**0.010**
Ts65Dn + Veh	Ts65Dn + 7,8-DHF	0.465	0.887	0.743	0.840	0.872
-		**Swim Speed (cm/s)**				
		**D1**	**D2**	**D3**	**D4**	**D5**
Eu + Veh	Eu + 7,8-DHF	0.632	0.953	0.689	0.485	0.976
	Ts65Dn + Veh	0.974	0.668	0.335	0.680	0.117
	Ts65Dn + 7,8-DHF	0.443	0.292	0.681	0.830	0.711
Eu + 7,8-DHF	Ts65Dn + Veh	0.617	0.627	0.179	0.277	0.123
	Ts65Dn + 7,8-DHF	0.225	0.267	0.431	0.382	0.732
Ts65Dn + Veh	Ts65Dn + 7,8-DHF	0.472	0.529	0.603	0.855	0.253

The numbers in bold correspond to statistically significant differences. Abbreviations: 7,8-DHF, 7,8-dihydroxyflavone; cm, centimeters; D, day; Eu, euploid; s, seconds; Veh, vehicle.

**Table 2:** p values of the Fisher LSD test for the indicated variables, during the learning phase of the MWM in euploid and Ts65Dn mice treated with vehicle or 7,8-DHF for 40 days, starting from 4 months of age.

-		**Latency (s)**							
		**D1**	**D2**	**D3**	**D4**	**D5**	**D6**	**D7**	**D8**
Eu + Veh	Eu + 7,8-DHF	0.296	0.135	0.170	0.281	0.078	0.051	0.293	0.209
	Ts65Dn + Veh	**0.017**	**0.006**	**0.001**	**0.003**	**0.022**	0.060	0.079	0.282
	Ts65Dn + 7,8-DHF	**0.018**	**0.005**	0.083	**0.002**	0.108	0.073	0.174	0.194
Eu + 7,8-DHF	Ts65Dn + Veh	0.283	0.331	**<0.001**	**0.001**	**0.001**	**0.001**	**0.015**	**0.037**
	Ts65Dn + 7,8-DHF	0.217	0.204	**0.008**	**<0.001**	**0.004**	**0.002**	**0.038**	**0.029**
Ts65Dn + Veh	Ts65Dn + 7,8-DHF	0.767	0.659	0.144	0.585	0.668	0.882	0.852	0.722
-		**Proximity (cm)**							
		**D1**	**D2**	**D3**	**D4**	**D5**	**D6**	**D7**	**D8**
Eu+Veh	Eu + 7,8-DHF	0.338	0.082	**0.005**	**0.016**	**0.004**	**0.018**	**0.012**	**0.006**
	Ts65Dn + Veh	**0.025**	0.097	0.424	0.389	0.539	0.538	0.345	0.840
	Ts65Dn + 7,8-DHF	0.882	0.660	0.349	0.541	0.834	0.640	0.747	0.883
Eu + 7,8-DHF	Ts65Dn + Veh	**0.006**	**0.003**	**0.001**	**0.003**	**0.001**	**0.006**	**0.002**	**0.005**
	Ts65Dn + 7,8-DHF	0.476	0.058	0.080	**0.009**	**0.016**	0.087	**0.050**	**0.020**
Ts65Dn + Veh	Ts65Dn + 7,8-DHF	**0.041**	0.316	0.117	0.889	0.470	0.332	0.268	0.754
-		**Swim speed (cm/s)**							
		**D1**	**D2**	**D3**	**D4**	**D5**	**D6**	**D7**	**D8**
Eu + Veh	Eu + 7,8-DHF	0.531	0.954	0.789	0.473	0.453	0.152	0.730	0.343
	Ts65Dn + Veh	0.620	0.292	0.821	0.749	0.815	0.929	0.654	0.689
	Ts65Dn + 7,8-DHF	0.018	0.104	0.150	0.913	0.616	0.119	0.484	0.071
Eu + 7,8-DHF	Ts65Dn + Veh	0.477	0.401	0.947	0.670	0.356	0.144	0.476	0.559
	Ts65Dn + 7,8-DHF	0.180	0.166	0.298	0.468	0.827	0.908	0.754	0.435
Ts65Dn + Veh	Ts65Dn + 7,8-DHF	0.432	0.480	0.225	0.706	0.494	0.113	0.292	0.152

The numbers in bold correspond to statistically significant differences. Abbreviations: 7,8-DHF, 7,8-dihydroxyflavone; cm, centimeters; D, day; Eu, euploid; s, seconds; Veh, vehicle.
